# A Review of the Establishment of Effective Conductive Pathways of Conductive Polymer Composites and Advances in Electromagnetic Shielding

**DOI:** 10.3390/polym16172539

**Published:** 2024-09-07

**Authors:** Xiaotian Nan, Yi Zhang, Jiahao Shen, Ruimiao Liang, Jiayi Wang, Lan Jia, Xiaojiong Yang, Wenwen Yu, Zhiyi Zhang

**Affiliations:** 1 College of Materials Science and Engineering, Taiyuan University of Technology, Taiyuan 030024, China; 2023510224@link.tyut.edu.cn (X.N.); theroch@163.com (Y.Z.); shenjiahao0044@link.tyut.edu.cn (J.S.); liangruimiao0307@link.tyut.edu.cn (R.L.); wangjiayi0234@link.tyut.edu.cn (J.W.); jialan@tyut.edu.cn (L.J.); 2 33rd Research Institute of China Electronics Technology Group Corporation, Taiyuan 030032, China; yxj--112233@163.com

**Keywords:** conductive polymer composites, electrical percolation threshold, conductive pathway, conductivity, electromagnetic interference shielding

## Abstract

The enhancement of the electromagnetic interference shielding efficiency (EMI SE) for conductive polymer composites (CPCs) has garnered increasing attention. The shielding performance is influenced by conductivity, which is dependent on the establishment of effective conductive pathways. In this review, Schelkunoff’s theory on outlining the mechanism of electromagnetic interference shielding was briefly described. Based on the mechanism, factors that influenced the electrical percolation threshold of CPCs were presented and three main kinds of efficient methods were discussed for establishing conductive pathways. Furthermore, examples were explored that highlighted the critical importance of such conductive pathways in attaining optimal shielding performance. Finally, we outlined the prospects for the future direction for advancing CPCs towards a balance of enhanced EMI SE and cost–performance.

## 1. Introduction

Continuous advances in information technology have led to the extensive utilization of electrical and electronic equipment in various domains, providing convenience for production processes and daily activities. However, such advances also cause issues in electromagnetic interference (EMI) [1,2,3,4]. On the one hand, EMI hinders the normal operation of electronic equipment, thereby causing the electronics to malfunction. On the other hand, it adversely affects human health and can also disrupt bioelectronic devices like cardiac pacemakers [5]. Therefore, given the rapid growth of radio frequency radiation sources and today’s high demand for reliable electronics, EMI shielding materials are needed to attenuate or cut off electromagnetic propagation. The electromagnetic shielding capability of a material is assessed by measuring its electromagnetic interference shielding efficiency (EMI SE) [6]. The conventional materials for high EMI SE are typically metal or metal-matrix composites, but these metals are difficult to process and easily corrode [7]. Conversely, conductive polymer composites (CPCs) have been extensively studied due to their excellent processability, low volume resistivity, and substantial EMI SE [4,8,9,10].

CPCs are composite materials consisting of a polymer matrix and dispersed conductive filler within the matrix [11,12]. The performance of CPCs is highly dependent on the type and content of the fillers (e.g., carbon−based, metallic, and conducting polymeric particles) [13,14,15]. Various fillers exhibit differing degrees of dispersion within the matrix, resulting in varying levels of difficulty in establishing conductive pathways. However, the volume resistivity will drop sharply only when effective conductive pathways are constructed using conductive fillers; specifically, the electrical conductivity increases dramatically by several orders of magnitude. The corresponding filler content is known as the electrical percolation threshold. By adjusting the structure and morphology of the conductive fillers, it is possible to form efficient conductive pathways at a lower electrical percolation threshold, thereby imparting conductivity to the insulated polymer matrix [16,17,18]. This construction of efficient conductive pathways is crucial for significantly enhancing EMI SE at lower filler content [19].

In this work, recent trends in the development of CPC are presented, including a discussion about the factors influencing the electrical percolation threshold of CPCs. It also details the methodology for constructing low−percolation conductive pathways and highlights examples that underscore the significance of such pathways in the field of electromagnetic shielding. Furthermore, projections are offered on the future development trends of CPCs in relation to electromagnetic shielding.

## 2. The Mechanism of Electromagnetic Interference Shielding

The mechanism of electromagnetic interference shielding can be explained by the widely recognized electromagnetic Schelkunoff’s transmission theory [20]. When an electromagnetic wave is transmitted onto the surface of a shielding material, a portion of it is reflected by the material’s surface, whereas the remaining portion penetrates into its interior. The material absorbs a fraction of the electromagnetic wave, and the remainder either undergoes multiple reflections within the material or continues to propagate through it towards the medium. The transmission process involves the division of attenuation into reflection loss (SE_R_), absorption loss (SE_A_), and multiple reflection loss (SE_M_). The total EMI SE is the aggregate of the three components, according to Schelkunoff’s theory on electromagnetic transmission. The formula is displayed below [11]:

(1) $$\mathrm{SE}\;={\;\mathrm{SE}}_{R}+{\;\mathrm{SE}}_{A}+{\;\mathrm{SE}}_{M}$$

(2) $${\mathrm{SE}}_{R}=20\log\frac{\eta_{0}}{4\eta_{s}}\;$$

(3) $$\eta_{s}=\sqrt{\frac{2\pi f\mu }{\sigma +2\pi f\varepsilon }}$$

(4) $${\mathrm{SE}}_{A}=20\log\;e^{d/\delta }$$

(5) $$\delta \;={\left(\sqrt{\pi f\mu \sigma }\right)}^{-1}$$

where *η_s_* is the intrinsic impedance of the shielding material, for conductive materials, σ >> 2πfε; therefore, Equation (3) simplifies to $\eta_{s}=\sqrt{\frac{2\pi f\mu }{\sigma }}$. *η_o_* is the EM incident wave propagating domain, for far-field radiation, *η_o_* is constant and is equal to 377 Ω. δ is the skin depth. Thus, the formula can be simplified as follows:

(6) $${\mathrm{SE}}_{R}=20\log\frac{\eta_{0}}{4}+20\log\frac{1}{\eta_{s}}=39.5+10\log\frac{\sigma }{2\pi f\mu }\;$$

(7) $${\mathrm{SE}}_{A}=8.7d\sqrt{\pi f\mu \sigma }$$

The formula incorporates the following variables: the thickness of the material (d), permeability (μ), conductivity (σ), frequency (f), and π signifying a constant. It can be seen that an augmentation in permeability results in a corresponding increase in absorption loss, whereas both reflection loss and absorption loss escalate with the increase in conductivity. 

For reflection loss, the inside of the shielding material should have effective charge carriers, requiring the material to have high conductivity; however, for multiple reflection loss, an adjustable structure such as a porous or layered configuration is required within the shielding material. For absorption loss, the shielding material should have electrical or magnetic dipoles to enhance electromagnetic dissipation [21]. The absorbed energy will be dissipated as heat [11]. In the process of attenuation, reflection loss and multiple reflection loss can lead to secondary pollution. Thus, enhancing the efficiency of absorption loss becomes the subsequent developmental direction for electromagnetic shielding materials. The loss mechanism reveals a positive correlation between good conductivity and EMI SE, suggesting that establishing efficient conductive pathways is an effective approach to enhancing CPC conductivity and electromagnetic shielding performance [11,21,22]. The factors influencing the electrical percolation threshold of CPCs are elaborated upon below.

## 3. Factors Influencing the Electrical Percolation Threshold in CPCs

The conductivity of CPCs is attributed to their ability to construct continuous conductive pathways within a polymer matrix through the inclusion of conductive fillers. These pathways are established when these fillers reach a specific concentration known as the electrical percolation threshold. As such, understanding CPC conductivity involves considering concepts like seepage theory and the electrical percolation threshold [23]. The relationship between the variation in the content of conductive fillers and the formation of conductive paths, as well as the impact on conductivity, can be elucidated as follows. When the filler content is low, the dispersion spacing of fillers in the matrix is large, impeding effective filler contact and resulting in the insulating properties of the composite material. With the increased addition of conductive fillers, the dispersion spacing decreases, facilitating enhanced fillers contact. Upon reaching the electrical percolation threshold, conductive pathways are constructed and conductivity sharply increases, transforming the composite material from an insulator into a semiconductor/conductor. The increase in conductivity tends to stabilize with further addition of fillers [24,25,26].

The factors influencing the electrical percolation threshold do not depend solely on the content of the conductive filler, but also on the shape and types of filler, as well as the composite matrix used. The variation in filler structure or the use of different conductive fillers can influence the threshold change for a given polymer matrix. Diverse types of conductive fillers, including metal powder [27,28], metal fiber [29,30], carbon−based fillers [31,32,33,34,35,36], etc. Metal-based fillers are characterized by their high cost and susceptibility to oxide layer formation, which can adversely affect the conductivity of materials. Meanwhile, carbon fiber, carbon nanotubes, graphene, and other carbon−based fillers offer advantages such as portability, a reduced agglomeration tendency, and high specific strength. Accordingly, these carbon−based fillers have extensive applications [23,37,38,39,40]. Lee et al. used a paste mixer to blend polydimethylsiloxane (PDMS) with three types of multiwall carbon nanotubes (MWCNTs) possessing different aspect ratios of 60, 173, and 400. They revealed that as the aspect ratio of MWCNTs increases, the electrical percolation threshold decreased while conductivity improves. MWCNTs possessing a higher aspect ratio have lower contact resistance because they have fewer contact points and higher electrical conductivity because they transport electrons on their own. Notably, polymer composites prepared using MWCNTs with an aspect ratio of 400 exhibit an EMI SE of at least 30 dB [41]. The composite material of polypropylene (PP) and high-structure carbon black was prepared by Al-Saleh et al. through the process of melt blending. They found an electrical percolation threshold of approximately 3 vol% [42]. Conversely, Zhao et al. prepared PP/segregated carbon black composite material with an electrical percolation threshold of 2.34 vol% [43], whereas the electrical percolation threshold of conventional PP/carbon black composites is typically 10 vol% or even higher [44]. The dispersion of conventional carbon black in the polymer matrix is challenging, resulting in a high electrical percolation threshold. In contrast, high-structure carbon black with its extensive specific surface area and irregular elongated chains facilitates the formation of effective conductive pathways. Segregated carbon black constructs distinctive conductive pathways within the matrix, demonstrating that the variation in conductive filler structure affects the construction of conductive pathways. In turn, the electrical percolation threshold is affected [45]. Wang et al. fabricated composites of polymethyl methacrylate (PMMA)/graphene and PMMA/chemically expanded graphite (CEG), revealing that the graphene sheets tend to aggregate, thereby impacting the continuity of the conductive pathways. However, CEG with a high porosity and a larger surface area can absorb a certain amount of dissolved PMMA, leading to a decreased number of polymer chain segments among CEG particles. Consequently, the likelihood for contact between the CEG particles increases, thereby facilitating the formation of an effective conductive pathway. Rheological analysis has demonstrated that incorporating CEG offers distinct advantages in constructing conductive pathways within polymer composites, with its electrical percolation threshold reaching as low as 0.29 vol% [22]. By establishing a collaborative electrical percolation effect model of carbon nanotube/graphene/polymer composites, Tang et al. demonstrated a synergistic effect between one-dimensional carbon nanotubes and two-dimensional graphene, resulting in reduced electrical percolation threshold of CPCs. This finding indicates that the combined utilization of carbon fillers with different distinct dimensions exerts a pronounced impact on the electrical percolation threshold of CPCs [46]. 

The threshold of electrical percolation varies between a single polymer matrix and two composite polymer matrices, even when the same conductive fillers are used. Yuan et al. prepared PP/CB and PE/CB composites using a single screw extrusion mechanism. The results indicated that the electrical percolation threshold of PE/CB composites is lower compared to than that of PP/CB composites. This finding can be attributed to the fact that polyethylene has fewer branch chains than polypropylene, which allows it to disperse more easily into the aggregated carbon black particles. As a result, the dispersion of carbon black in the matrix is promoted [47]. Huang et al. discovered that MWCNTs more easily construct conductive pathways in poly(epsilon−caprolactone) (PCL) than isotactic polypropylene (iPP). The dispersion of MWCNTs in a PCL matrix is enhanced due to the lower viscosity of the PCL matrix and the strong interaction between the PCL segment and MWCNTs [48]. Mao et al. prepared a blend of polystyrene (PS)/polymethyl methacrylate (PMMA)/octadecyl lamine−functionalized graphene (GE-ODA) through solution mixing. In the composites with a mass ratio of PS to PMMA at 1:1, a co−continuous structure was formed by the PS phase and PMMA phase. By selectively positioning and permeating GE-ODA nanosheets into the PS phase, the composites exhibit favorable electrical properties, resulting in decreased threshold of conductive percolation from 2 wt.% to 0.5 wt.% compared with the original single polymer composition [49]. The double percolation structure is achieved through the selective distribution of conductive fillers throughout the incompatible polymer matrix, wherein the conductive fillers achieve percolation in one phase of the polymer (first percolation), while the polymer matrix forms a continuous phase to achieve percolation (second percolation) [25].

The concept of double percolation has been initially proposed by Sumita et al., who observed an uneven distribution of carbon black in each component of the polymer blend. In one case, the fillers are predominantly distributed within a specific phase of the blended matrix, exhibiting a relatively uniform distribution similar to that observed in single polymer composites. In the other case, the filler distribution is concentrated at the interface between the two polymers. To explain these findings, Sumita et al. put forth a theoretical model known as Young’s equation, which is presented below [50]:

(8) $$\omega_{a}=\frac{\gamma_{stuff-B}\;\;-\;\;\gamma_{stuff-A}}{\gamma_{A-B}}$$

where ω_a_ represents the wetting coefficient; *γ_A−B_* denotes the interfacial tension between polymer A and polymer B; *γ_stuff−B_* refers to the interfacial tension between polymer B and the filler; and *γ_stuff−A_* signifies the interfacial tension between polymer A and the filler. For ω_a_ < −1, the filler is primarily distributed in polymer B. For ω_a_ > 1, the filler is primarily distributed in polymer A. For −1 < ω_a_ < 1, the filler is predominantly located at the phase interface. The non-uniform dispersion of conductive fillers significantly affects the conductivity of CPCs and can effectively lower the electrical percolation threshold.

## 4. The Methodology for Achieving Effective Conductive Pathways

Some conductive fillers may require a relatively high filling amount to construct effective conductive pathways, which is characterized by a high electrical percolation threshold. Although the polymer composites have conductivity, it will have a significant impact on the mechanical strength of the polymer composites and result in increased costs. Moreover, at a high packing concentration, the conductive particles tend to agglomerate, which can negatively impact the formation of a well−structured conductive pattern [51]. The presence of a high filler content leads to the agglomeration of the conductive filler, thereby impacting the formation of a continuous conductive pathways [52,53]. Therefore, researchers are increasingly focusing on CPCs with a low electrical percolation threshold. Traditional filler treatment, the cooperative utilization of fillers, and the design of double percolation pathway structures are all methods that can be used to achieve the electrical percolation threshold.

### 4.1. Conductive Filler with Metal Coating

**Figure 1:**
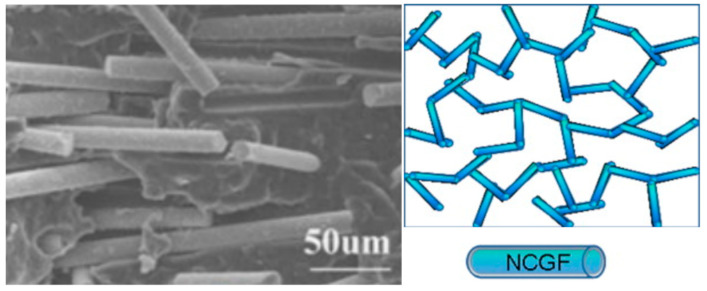
Schematic representation of the conductive pathways created by nickel-coated glass fibers within a composite material [55].

By subjecting non−conductive fillers to a metal coating treatment, they can acquire conductive properties. For materials like carbon fibers that already possess inherent conductivity, this treatment enhances their conductive characteristics. Xing et al. discovered that the application of nickel plating treatment on a filler leads to the formation of a conductive nickel layer on its surface, thereby facilitating electron transport and enhancing the conductivity of composite materials [54]. Guo et al. conducted a study in which they treated glass fiber with NiCl_2_·6H_2_O, plated its surface with nickel layer, and prepared a PP/nickel−coated glass fibers (NCGFs) mixture by solution mixing method. The results of their research show that the electrical percolation threshold of PP/NCGFs composites is 0.46 vol%, and the conductivity reaches 8.7 S/cm. As shown in Figure 1, nickel-plated glass fibers make contact with one another to construct well-conductive pathways [55]. Bryant et al. blended NCCF and long fiber nickel-coated carbon fiber (LFNCCF) with PC separately and the findings demonstrated that EMI SE of PC/NCCF blends containing 20 wt.% NCCF reached up to 90 dB. Additionally, PC/LFNCCF blends reached 90 dB EMI SE with just 10 wt.% LFNCCF [56]. Shui et al. highlighted the critical role of nickel filaments in significantly boosting the EMI shielding performance of polymer composites, even at relatively low volume fractions. They electroplated nickel on carbon filaments to prepare nickel filaments with a diameter of only 0.4 microns, and mixed them with polyether sulfone (PES), achieving an EMI SE of 91.7 ± 6.6 dB at a 19% volume fraction of the nickel filaments [57,58]. In their other work, nickel filaments were mixed with silicone rubber and pressed to prepare composites with an EMI SE of 90.5 ± 5.5 dB [59].

### 4.2. Synergistic Effect of Multiple Fillers

**Figure 2:**
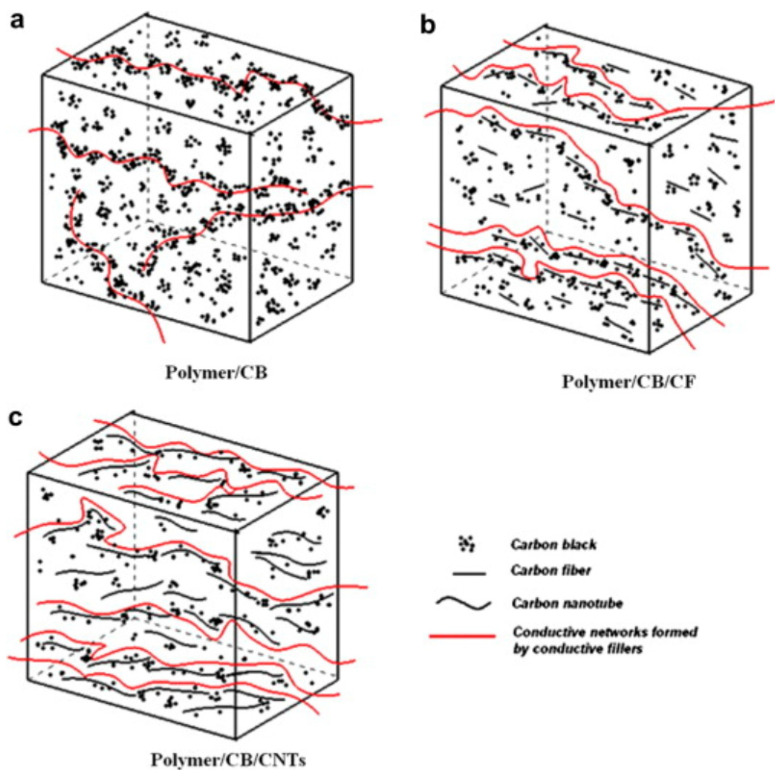
The schematic diagram illustrates three distinct types of conductive pathways. (**a**) the conductive pathways formed by CB in polymer; (**b**) the conductive pathways formed by CB and CF in polymer; (**c**) the conductive pathways formed by CB and CNTs in polymer [62].

The use of a single filler in conductive materials can often limit the construction of effective conductive pathways due to its inherent properties and structure. However, utilizing different carbon-based conductive fillers in combination can promote the construction of more efficient pathways for conductivity [60,61]. Zhao et al. incorporated carbon fiber as a secondary filler to fabricate composites of PP/CB/CFs. The inclusion of carbon fiber serves as bridging conductive pathways within the matrix, resulting in a reduction in the electrical percolation threshold from 2.34 vol% observed in single-component PP/CB composites to 0.785 vol% [43]. Wen et al. conducted a study in which they prepared PP/CB, PP/MWCNT, and PP/CB/MWCNT systems through melt blending. They revealed that the electrical percolation threshold of the PP/CB/MWCNT system is lower than that of the first two systems when subjected to multistage tensile extrusion. This synergistic effect results in reduced resistivity. The multistage stretch extrusion process applies strong shearing or stretching forces to the melt, thereby enhancing the filler dispersion and orientation. In addition, the study also elucidated the differences in conductive pathways constructed by various fillers within the polymer matrix (Figure 2). CB exhibits a grape-like aggregate shape with limited conductive pathways (Figure 2a). The conductive pathways created by the combination of CB and CF resemble a grape-cluster structure, where the CB particles act as grapes and CF serves as branches (Figure 2b). Similarly, the mixture of CB and MWCNT leads to grape cluster-shaped conductive pathways (Figure 2c) [62]. Chen et al. have demonstrated, through rheological theory, that composites of iPP/CB/MWCNTS exhibit a lower electrical percolation threshold and superior electrical properties compared with composites containing only a single filler [63].

### 4.3. Directional Dispersion of Fillers

**Figure 3:**
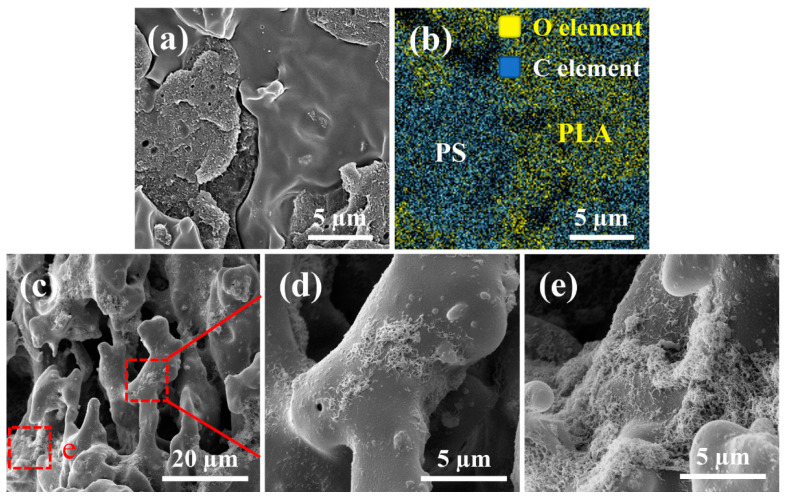
SEM images of PLA/PS/MWCNT blends loading 3 wt.% MWCNT annealed in 160 °C and 20 MPa CO_2_ for 1 h: (**a**) image; (**b**) C and O EDS elemental mapping of (**a**). SEM images of PS domain extracted using cyclohexane: (**c**) image; (**d**,**e**) are the part magnification of (**c**) [64].

**Figure 4:**
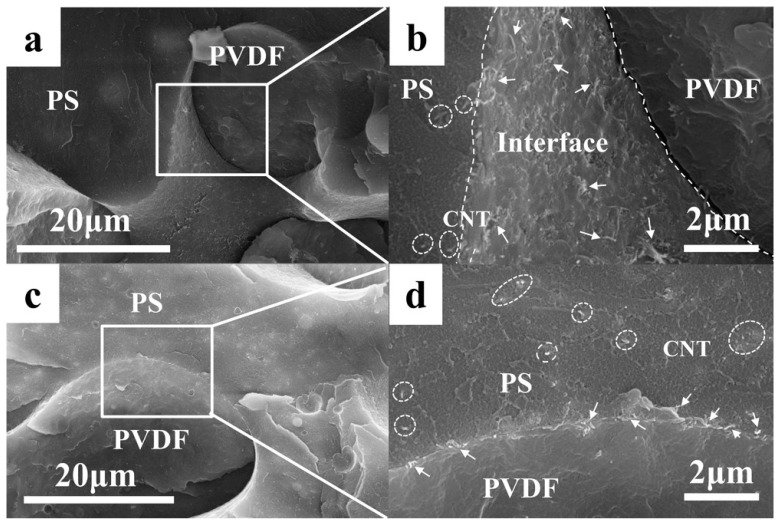
The phase morphology of PVDF/PS/CNTs-PMMA composites with different contents of CNTs-PMMA. (**a**,**b**) 0.36 vol%, (**c**,**d**) 0.68 vol%. The dashed lines indicate the two-phase interface, the white arrows indicate CNTs-PMMA distributed at the interface, and the circles indicate CNTs-PMMA were located in the PS phase [67].

To achieve a co-continuous structure, Zuo et al. fabricated a composite material comprising polylactic acid (PLA), polystyrene (PS), and MWCNTs with a PLA to PS mass ratio of 1:1, and the composite material is then annealed in supercritical carbon dioxide (scCO_2_). Due to the plasticizing effect of scCO_2_ on the polymer matrix, both polymers exhibit a significant reduction in viscosity, leading to coarsening the blend phase interface and thus facilitating the formation of conductive pathways. MWCNTs dispersed a few on the surface of the PLA phase (Figure 3d), and mostly at the walls of holes which were caused by the extraction of PS (Figure 3e). Consequently, the electrical percolation threshold decreases from 0.31 wt.% to 0.16 wt.%, accompanied by increased EMI SE from 32.8 dB to 37.4 dB. The formation of double percolation pathways structure is achieved prior to annealing [64]. In another study conducted by Zuo et al., the electrical percolation threshold of PCL/PS/MWCNTs composites is reduced by 50% after scCO_2_ annealing treatment. Additionally, the EMI SE of the composites with varying MWCNTs content was observed increasing following scCO_2_ treatment [65]. Liu et al. fabricated PLA/polycaprolactone (PCL)/MWCNTs composites through melt blending, with PLA serving as the masterbatch prepared by incorporating SiO_2_. They demonstrated that the addition of SiO_2_ enhances the viscosity and elasticity of the PCL phase, leading to a transition in morphology from segregation into a continuous phase. MWCNTs are selectively dispersed within the PCL phase, resulting in a double percolation structure. This unique structure enables the PLA/PCL/MWCNT nanocomposites to achieve an exceptionally low electrical percolation threshold of 0.06 vol% [66]. Yang et al. conducted in situ polymerization to prepare CNTs−PMMA, and subsequently fabricated composites of polyvinylidene chloride (PVDF)/PS/carbon nanotubes (CNTs) and PVDF/PS/CNTs-PMMA through melt blending. The results of the study demonstrated that both the PVDF phase and the PS phase exhibited a bi-continuous structure, with the modified CNTs being distributed within the PS phase. The introduction of PMMA (poly(methyl methacrylate)) grafting onto the surface of CNTs leads to enhanced compatibility between CNTs and the PVDF phase. Consequently, CNTs−PMMA are selectively distributed at the interface between these two phases, as depicted in Figure 4. The electrical percolation threshold of PVDF/PS/CNTs−PMMA nanocomposites is determined to be 0.07 vol%, which is significantly lower by 50% compared to that of PVDF/PS/CNT nanocomposites [67].

Tu et al. utilized the HAAKE extruder to prepare composites of PP/PE/thermally reduced graphene oxide (TRG) and (PP/TRG)/PE. The former involves the simultaneous melting and blending of PP, PE, and TRG in an extruder, whereas the latter uses a two-step processing method where a PP/TRG masterbatch is first prepared and then blended with PE. The findings reveal that in the PP/PE/TRG composites, TRG exhibits selective dispersion within the PE phase. Conversely, in the (PP/TRG)/PE composites, TRG is located at the interface between PP and PE blends. Notably, the electrical percolation threshold for (PP/TRG)/PE composite ranges from 0.5 wt.% to 1 wt.%, which is lower than that observed for PP/PE/TRG composites [68]. Strugova et al. conducted prepared PP/PS/MWCNT composites by using a two-step method. First, a PP/MWCNT masterbatch is prepared, followed by blending it with PS. The effects of three different heat treatments (fast cooling, isothermal treatment, and slow cooling) on the electrical percolation threshold of the composites are compared and analyzed. The results show that the electrical percolation threshold caused by isothermal and slow cooling treatment is significantly lower. This study reduces the electrical percolation threshold resulting from the co−continuous morphology of PP/PS/MWCNT composites and achieves an ultra-low electrical percolation threshold through heat treatment [69]. The double percolation structure can be achieved through the surface modification of the conductive filler and adjustment of the viscoelasticity of the polymer matrix. Furthermore, heat treatment can promote phase interface coarsening in this structure, thereby enhancing the dispersion of the conductive filler. Compared with CPCs based on a single polymer, the double percolation pathways structure design facilitates the selective positioning of conductive filler. Researchers prefer the conductive fillers to be evenly distributed at the interface of the immiscible polymer mixture, and a minute quantity of conductive filler is utilized to construct conductive pathways at the continuous interface. The outcome is a reduced threshold for electrical percolation [23,70].

## 5. Advances in CPCs in Electromagnetic Shielding Field

**Figure 5:**
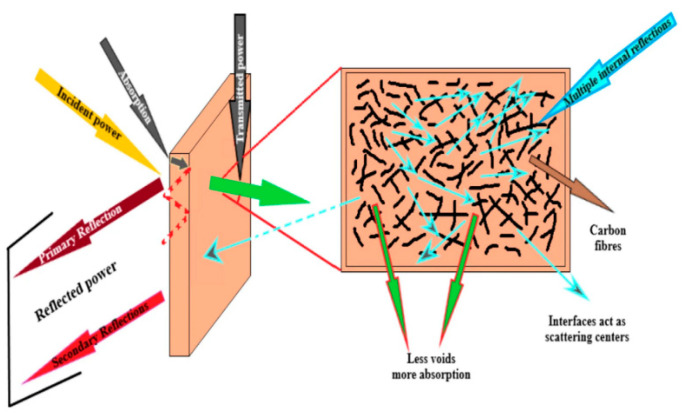
Diagram of EMI shielding mechanism in CF-filled PP composites [71].

**Figure 6:**
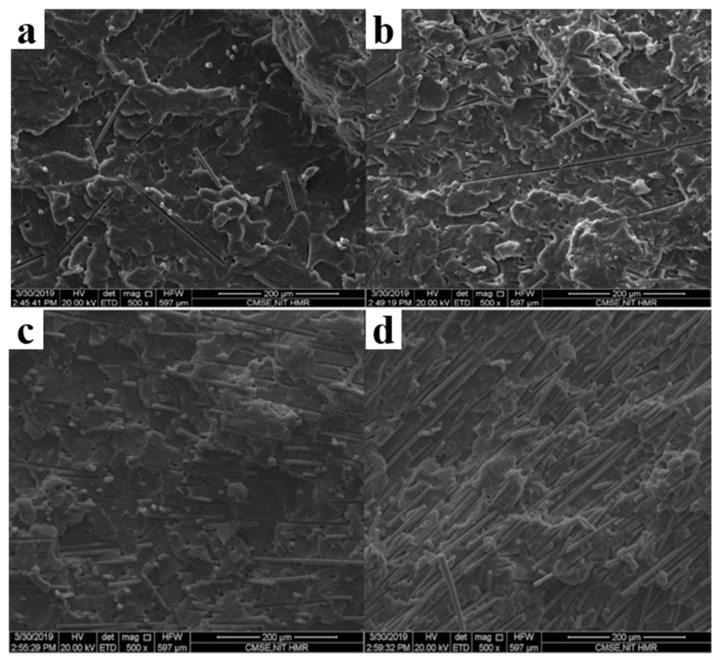
SEM images of (**a**) 5 wt.%, (**b**) 10 wt.%, (**c**) 15 wt.%, and (**d**) 20 wt.% CF/PP composites [71].

The conductivity and electromagnetic shielding performance of CPCs can be enhanced by constructing the efficient conductive pathways with a low electrical percolation threshold. Kaushal et al. described in detail the EMI shielding mechanism of CFfilled PP, as depicted in Figure 5, with the SEM images depicted in Figure 6. The reflection loss arises from the interaction between electromagnetic waves and charge carriers, namely electrons and holes, present on the material’s surface. Absorption loss occurs due to the interaction between electric dipoles and electromagnetic fields during EMI. The highest EMI SE achieved by the prepared PP/CF composite is 32.92 dB [71]. In another study, Kaushal et al. prepared PP/CB/CF composites by using twin screw extruder melt blending and investigated their conductivity. They revealed that the electrical percolation threshold is less than 5 wt.%, with a resulting conductivity of 2.31 × 10^−4^ S/cm. Their study highlights that the rapid changes in conductivity are attributed to the formation of continuous conductive pathways known as the electrical percolation pathways. The EMI SE is also tested, and the absorption and reflection losses are compared. The results indicate that the EMI SE increases proportionally with the increased content of conductive filler. It can reach a value of 44.43 dB in the X−band frequency range, and the absorption loss is dominant [72]. Lecocq et al. prepared PP/CNT polymer composites through melt blending, with an electrical percolation threshold of 0.49 vol%. With increased filler content, both conductivity and EMI SE significantly improve, reaching conductivity values of up to 20 S/m and EMI SE values of up to 90 dB. EMI SE in the X−band and Ku-band frequency range is found to be excellent [73]. The aforementioned research demonstrates that the selection and collaborative utilization of fillers impact the formation of conductive pathways, so the establishment of efficient conductive pathways are advantageous in enhancing the EMI SE of CPCs.

**Figure 7:**
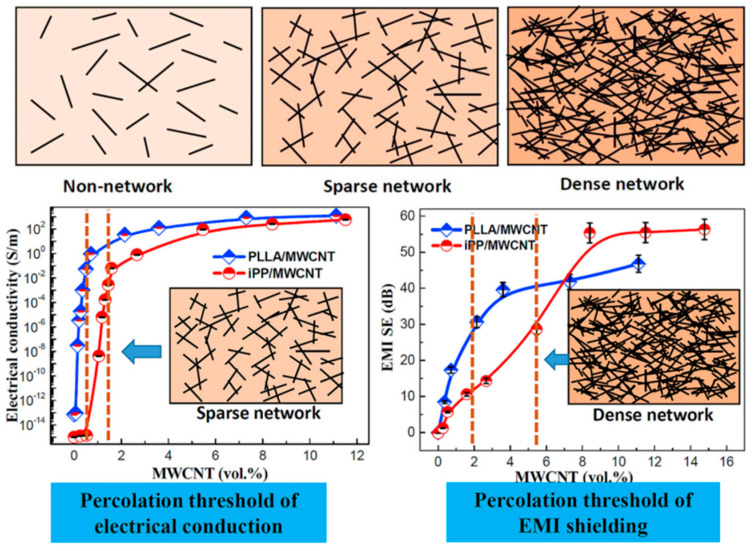
Conductivity and EMI SE of PLLA/MWCNT and iPP/MWCNT nanocomposites with varying MWCNT contents, along with the corresponding pathways to achieve threshold values, the dashed lines represents the percolation threshold [74].

**Figure 8:**
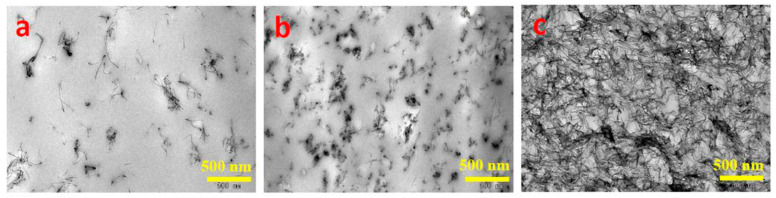
TEM (**a**–**c**) images of the PLLA/MWCNT nanocomposites with 0.07 (**a**), 0.36 (**b**), and 2.14 (**c**) vol% MWCNT [74].

**Figure 9:**
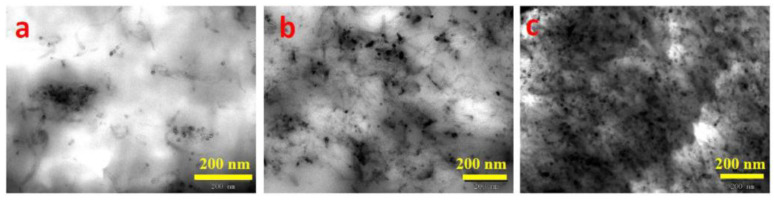
TEM (**a**–**c**) images of the iPP/MWCNT nanocomposites with 0.26 (**a**), 1.58 (**b**), and 5.46 (**c**) vol% MWCNT [74].

iPP/MWCNT nanocomposites and L-polylactic acid (PLLA)/MWCNT nanocomposites were prepared by Shi et al.; the EMI shielding threshold and electrical percolation threshold of the former are approximately 5.40 vol% and 1.40 vol%, respectively. However, due to the superior dispersion of MWCNTs in PLLA, the latter exhibits reduced EMI shielding percolation threshold and electrical percolation threshold values of approximately 2.00 vol% and 0.40 vol%. The determination of the electrical percolation threshold and EMI shielding percolation threshold relies on three types of pathway structures, namely, unformed pathways, sparse pathways, and dense pathways. As depicted in Figure 7, Figure 8 and Figure 9, achieving high conductivity can be accomplished by constructing sparsely distributed MWCNT pathways. Dense MWCNT pathways are required for attaining high-performance EMI SE [74]. Huang et al. also confirmed this phenomenon [48]. The measurement and prediction of the electrical percolation threshold and EMI shielding percolation threshold are crucial in practical applications. Insufficient filler content results in inadequate electrical properties and electromagnetic shielding capabilities, whereas excessive filler content leads to high costs and compromised mechanical properties of composite materials. Therefore, the prediction of the percolation threshold allows for achieving high EMI SE performance with a lower filler content. This approach is highly cost−effective for the preparation of composite materials with superior performance.

**Figure 10:**
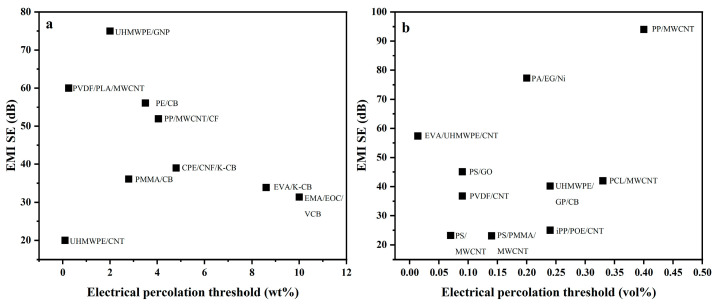
The electrical percolation threshold values and maximum EMI SE for CPCs, mass fraction (**a**), volume fraction (**b**).

**Figure 11:**
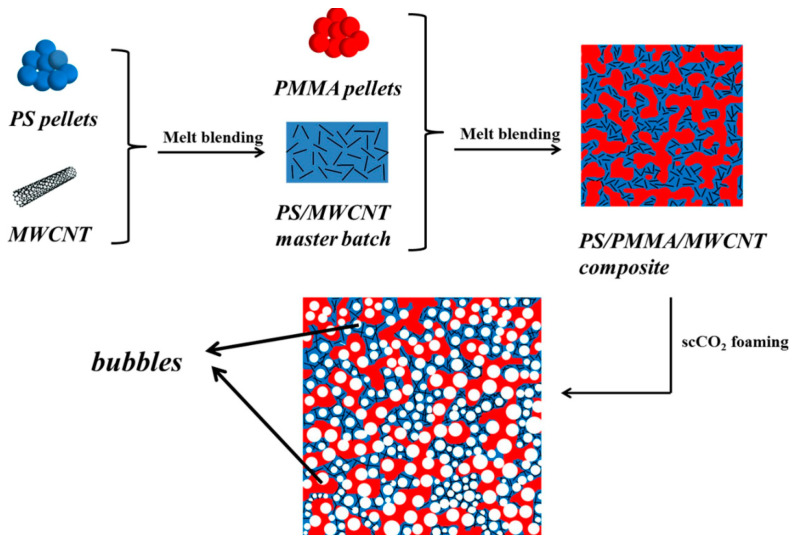
Schematic of the fabrication procedure of porous PS/PMMA/MWCNT composites with double-percolated structure [115].

We have summarized the CPCs used for EMI shielding, along with the processing methods, carbon−based filler content, electrical percolation threshold, conductivity, and EMI SE for the composites in Table 1 [32,48,56,57,59,64,73,75,76,77,78,79,80,81,82,83,84,85,86,87,88,89,90,91,92,93,94,95,96,97,98,99,100,101,102,103,104,105,106,107,108,109,110,111,112,113,114,115,116,117,118,119,120,121,122,123]. The CPC systems generally transition from an insulator to a conductor at a very low conductive filler loadings (below 0.45 vol% or 15 wt.%). However, there is no consensus on the exact electrical percolation thresholds, as they vary with different types of conductive fillers and matrices. Figure 10, based on the data presented in Table 1, shows that compared to CB, high-aspect-ratio carbonaceous materials exhibit lower electrical percolation thresholds and higher EMI SE. To achieve a high EMI SE, effective dispersion of these high-aspect-ratio carbonaceous materials is essential. Another crucial parameter of CPCs is the inherent electrical conductivity of the conductive fillers, which is related to the band gap [124]. Notably, the nickel-coated carbon filaments are excellent candidates for high-performance EMI shielding materials due to their superior electrical conductivity, ferromagnetic properties, and high magnetic and dielectric loss tangents [110,125,126]. Moreover, the uniform distribution of the nickel coating further optimizes its performance in practical applications. To prepare CPCs, various processing methods are employed. Melt blending is the main processing method using screws, with the temperature of the screw typically set to match the processing temperature of the polymer matrix [48,127,128]. Solution mixing, or wet-mixing, involves dissolving the polymer matrix in an organic solvent and adding the fillers, followed by solvent evaporation to obtain the blend [97,129,130]. Lanticse et al., using the doctor blade technique, also known as the tape casting technique, prepared a polymer composite with preferentially aligned multiwalled carbon nanotubes. The results indicate that the conductivity of the nanotubes exhibits anisotropy due to their preferential alignment, with higher conductivity observed parallel to the blade compared to the vertical direction [131]. The blends obtained through the two methods are subsequently subjected to injection [75] or hot-pressing techniques [82] in order to obtain the test sample. Many studies have utilized other processing methods, such as foaming, to fabricate composites with a porous structure [90]. This approach has been shown to result in a lower percolation threshold and improved EMI SE of the composite materials [76,115,132,133]. Chen et al. prepared PS/PMMA/MWCNT composites by two-step melt blending, which was subsequently subjected to scCO_2_ foam environmentally friendly microporous molding technology for the introduction of a microporous structure. After foam molding, the electrical percolation threshold of the composites was reduced from 0.18 vol% to 0.14 vol%, and the EMI shielding performance was improved from 37.79 to 57.7 dB·cm^3^/g. The processing method is shown in Figure 11 [115].

## 6. Challenge

Advances have led to the creation of composites with impressive EMI SE at relatively low filler contents. This has been achieved through the strategic coordination of multiple fillers and the blending of various polymer substrates, which lowers the percolation threshold. However, challenges remain.

Achieving the uniform dispersion of conductive fillers, such as CNTs, graphene, or metal nanoparticles, within a polymer matrix remains a significant challenge. Despite the development of advanced techniques like chemical functionalization, the grafting of polymer chains onto filler surfaces, and the application of coatings to enhance filler dispersion and improve interfacial bonding, these methods are still not fully optimized for large-scale production and often yield inconsistent results across different studies due to the lack of uniform standards.

While exceeding the percolation threshold can increase conductivity, it can also lead to brittleness, increased viscosity during processing, and challenges in maintaining structural integrity. Conversely, insufficient filler content fails to establish an effective conductive network, resulting in poor EMI shielding. It is essential to achieve an optimal filler concentration that is high enough to ensure efficient EMI shielding while maintaining the mechanical properties necessary for the material’s intended application. Methods like using electrospinning nanofibers within the polymer matrix can create highly efficient conductive pathways. By aligning the nanofibers, it is possible to enhance conductivity even at lower filler contents, potentially reducing the risk of brittleness and processing challenges.

The current theoretical model regarding the construction of conductive pathways and the determination of the EMI SE often differs from practical observations. Therefore, more robust theories incorporating multi-scale simulations that bridge the gap between molecular-scale interactions and macroscopic material properties are required to offer theoretical guidance on the relationship between the construction of conductive pathways and EMI SE in CPCs.

Moreover, CPCs should not only focus on electromagnetic shielding materials, their muti-functional development should also be considered, combining electrical conductivity and EMI shielding with additional properties, such as thermal conductivity, flame retardancy, self-healing, or shape memory. These multi-functional materials are particularly valuable in advanced electronics, aerospace, and automotive applications. Additionally, functional devices with electromagnetic shielding properties have rarely been observed in the available literature. MXene, with its excellent electrical conductivity, hydrophilicity, and flexibility, shows significant potential for advancements in areas such as intelligent sensing and wearable heaters [134,135]. Such devices could open up new possibilities for CPCs, expanding their applications and making them even more relevant in high-tech industries.

However, the processing of multi-component CPCs remains complex and costly, posing challenges for mass production. The high complexity and cost hinder large-scale manufacturing, making it difficult to bring these advanced materials into widespread commercial use. Therefore, optimizing the balance between cost and performance, especially in terms of filler content and EMI shielding, is of paramount importance. Several strategic approaches can be employed to achieve this balance.

Selecting cost-effective filler and advanced manufacturing techniques: The choice of filler materials plays a critical role in balancing the cost and performance. By choosing fillers that offer high conductivity at lower concentrations, such as CNTs over conventional fillers like CB, manufacturers can reduce material usage and costs. Techniques like electrospinning and 3D printing further enhance filler distribution and alignment within the polymer matrix, creating efficient conductive networks at lower filler loadings, improving composite performance while minimizing material usage.

Using hybrid fillers and optimizing its concentration: Combining high-performance nanofibers (carbon nanofibers or nickel filaments) with more economical conventional fillers can optimize both cost and performance. This approach can achieve the required EMI shielding and mechanical properties without significantly increasing costs. The hybrid approach allows for the advantages of both filler types, balancing conductivity, mechanical strength, and cost. Additionally, identifying the minimum filler concentration needed to meet performance requirement is vital. Techniques such as theoretical model or machine learning can predict optimal concentrations, ensuring cost-effective production while maintaining necessary properties.

Recycling and reuse of fillers: Incorporating recycled or reused fillers can lower costs while maintaining performance. Developing reclamation methods for fillers from waste or end-of-life products contributes to more sustainable and cost-effective production processes. This approach also aligns with increasing demand for environmentally friendly materials and processes.

Finally, tailoring the composite material to the specific requirements of the application can help in optimizing the balance between cost and performance. For applications where high EMI shielding is critical, a higher investment in high-performance fillers may be justified. In contrast, for less demanding applications, a more cost-effective approach with lower filler content might be appropriate. Understanding the end-use environment and required performance characteristics allows for more informed decisions about the trade-offs between cost and performance. By carefully considering these strategies, manufacturers can achieve a well-balanced composite material that meets performance requirements while keeping costs under control.

## 7. Summary and Prospects

CPCs, as the functional material, exhibit exceptional machining properties, electrical conductivity, and electromagnetic shielding capabilities. These composites hold promising potential in various applications such as electronic component protection, base station shielding, and aerospace industries. Researchers are primarily focused on achieving a low electrical percolation threshold, constructing efficient conductive pathways, and attaining good EMI SE. Despite extensive application, the mechanism of EMI shielding for CPCs remains unclear. Therefore, more robust theories are required to offer theoretical guidance on the construction of conductive pathways under various fillers and polymer substrates in CPCs. Further exploration into the relationship between electric percolation threshold and EMI SE is warranted. Such studies could enhance the durability of CPCs and broaden their application in flexible wearable devices, particularly for workers operating in high EMI environments. Additionally, establishing theoretical models to analyze the relationship between reflection loss and absorption loss in CPCs for improving the efficiency of electromagnetic shielding materials, which in turn reduces the secondary pollution, aligns with the call for green development. By optimizing the balance between cost and performance, the objective of achieving superior EMI SE using a low content of conductive filler can facilitate the mass production and extensive dissemination of these materials.

## Figures and Tables

**Table 1:** Summary of polymers, carbon−based fillers, processing methods, filling quantities, electrical percolation threshold, conductivity, and EMI SE.

	Polymers	The Type of Carbon-Based Fillers	Processing Methods	Filler Content	Electrical Percolation Threshold	Conductivity	EMI SE
[73]	Polypropylene (PP)	MWCNT Average diameter: 9.5 nm length: 1.5 μm	Melt blending Hot pressed	0.25–7.5 vol%	0.4 vol%	580 S/m	94 dB
[48]	PP	MWCNT Average diameter: 9.5 nm length: 1.5 μm	Melt blending Compression molding	~0–11 vol%	1.24 vol%	~100 S/m	28.6 dB
[75]	PP	MWCNT Average diameter: 20–30 nm Average length: 0.5–2 μm Carbon Fiber (CF) Length: 6 mm	Melt blending Injection molding	0.5 wt.% 0–20 wt.% 0–20 wt.%	4.05 wt.%	9.58 × 10^−2^ S/cm	51.9 dB
[76]	PP	CB Diameter: 30–45 nm CNT Length: 10–30 μm Diameter: 8 nm (1/1)	Compression molding Structural manipulation Solid-state ScCO_2_ foaming	05 wt.%	0.016 vol%	~6.67 × 10^−3^ S/cm	72.23 dB·cm^3^/g
[77]	PP	Nickel-coated carbon fiber Diameter: 7 μm	Melt blending Injection molding	10–30 wt.%		10^1^–10^2^ S/cm	48.4 dB
[78]	Copolymerization Polypropylene (co−PP)	CNT Average diameter: 9.5 nm Length: 1.5 μm	Melt blending Injection molding	0–3.5 wt.%		14.4 S/m	32 dB
[56]	Polycarbonate (PC)	Long fiber nickel-coated carbon fiber Length: 12 mm	Pultrusion process Cube Blending	20 wt.%			100–110 dB
[79]	Polyethylene (PE)	CB	Solution-mixture	0–10 wt.%	3.5 wt.%	~10^−2^ S/cm	56.1 dB
[80]	PE	Segregated carbon nanotube (s−CNT)	Compression molding	0–5 wt.%	0.013 vol%	~50 S/m	~50 dB
[81]	Chlorinated Polyethylene (CPE)	Carbon nanofiber Length: 30–100 μm diameter 70–200 nm Ketjen carbon black (K-CB) Particle size: 50–60 nm (1/1)	Solution blending	0–15 wt.%	4.8 wt.%	0.022 S/cm	37–39 dB
[82]	Ultrahigh Molecular Weight Polyethylene (UHMWPE)	CNT Average diameter: 9.5 nm Length: 1.5 μm	Wet-mixing Hot compression	0–10 wt.%	0.096 wt.%	~1 Ω·cm	20 dB
[83]	UHMWPE	CNT Average diameter: 9.5 nm Average length: 1.5 μm	Solid phase extrusion	0–4 wt.%	0.085 vol%	~10 S/m	~37 dB
[84]	UHMWPE	Graphite platelets (GP) Lateral dimension: 20 μm CB Average particle size: 25 nm (1/1)	Hot compression	0.1–15 wt.%	0.24 vol%	33.9 S/m	40.2 dB
[85]	UHMWPE	Carbon nanostructures (CNS)	Compression molding	0–10 wt.%	0.48 wt.%	~1 S/cm	60.7 dB
[86]	UHMWPE	Graphene nanoplatelets (GNP)		0–40 wt.%	2–3 wt.%	0.11 Ω·cm	~75 dB
[87]	Polycaprolactone (PCL)	MWCNT Average diameter: 9.5 nm Length: 1.5 μm	Solution-mixture Compression molding	0–0.15 wt.%	~0.016 wt.%	~2.49 × 10^−2^ S/cm	~23.8 dB
[48]	PCL	MWCNT Average diameter: 9.5 nm Length: 1.5 μm	Melt blending Compression molding	~0–11 vol%	0.33 vol%	~100 S/m	42 dB
[88]	PCL	MWCNT Average diameter: 9.5 nm Length: 1.5 μm	Melt blending Compression molding layer by layer	5–15 wt.%			61.5 dB
[89]	PCL	MWCNT Average outer diameter: 10 nm	Co−precipitation ScCO_2_ foaming	0.049–0.249 vol%			60–80 dB
[90]	Polystyrene (PS)	MWCNT Mean diameter: 9.5 nm Mean length: 1.5 μm	High-speed mechanical mixing Supercritical carbon dioxide (scco2) foaming	0–1.88 vol%	0.07 vol%	8.05 S/m	23.2 dB
[91]	PS	Graphene oxide (GO) Thickness: 1–2 nm Average particle size: 0.87 μm	Solution-mixture Solid-phase compression molding	0–3.47 vol%	0.09 vol%	43.5 S/m	45.1 dB
[92]	PS	MWCNT	Melt blending Compression molding	0–20 wt.%	~4 wt.%	~0.1 S/cm	~60 dB
[93]	Polyamide 6 (PA6)	Expanded graphite (EG) 0.15 vol% NiCl2·6H2O	Electroless plating Hot compression	0–0.9 vol%	0.2–0.3 vol%	778 S/m	77.3 dB
[94]	PA6	PAN-based carbon fiber Nickel powders	Melt blending Thermoplastic pultrusion	0–14 vol% 0–10 wt.%	5 vol%	10^−2^–10^−3^ S/m	~36 dB
[95]	PA12	Nano-carbon black (CB)	Selective laser sintering	0–8 wt.%	0.87 wt.%	~10^−1^ S/m	
[96]	Polyvinylidene Fluoride (PVDF)	CNT Average diameter: 9.5 nm Length: 1.5 μm	Solid-phase extrusion	0–4 wt.%	0.09 vol%	74.5 S/m	36.8 dB
[97]	PVDF	CNT Graphene	Solution-mixture	5 wt.% 10 wt.%			27.58 dB
[98]	PVDF	MWCNT Average diameter: 40–60 nm Length: 0.5–40 μm	Solution casting	0–5 wt.%			35 dB
[32]	PVDF	GNP	ScCO_2_ foaming Solution-mixture	4 wt.%			~50 dB
[99]	Polydimethylsiloxane (PDMS)	GN	Chemical vapor deposition	1.2 wt.		6100 S/m	90 dB
[100]	PDMS	SWCNTs Diameter: ~2–4 nm, Length: ~30 μm	Sol–gel self-assembly method	0.25–0.35 wt.%		1.2 S/cm	31 dB
[101]	PDMS	Graphene layers MWCNTs Diameters: 60–80 nm Length: ~20 μm	Chemical vapor deposition Thermal curing and Ni etching	0.28–2.7 wt.% 0–5 wt.%		31.5 S/cm	~75 dB 833 dB·cm^3^/g
[102]	Poly (Methyl Methacrylate) (PMMA)	CB Surface area: 1400 m^2^/g	Hot compression Solution-mixture	0–10 wt.%	2.79 wt.%	2.2 × 10^−3^ S/cm	36.1 dB
[103]	PMMA	GNP Thickness: 3–10 nm Average platelet diameter: 5–10 μm MWCNT Diameter: 30–50 nm Length: 10–20 μm	Solution-mixing ScCO_2_ foaming	1.5–4 wt.% 3–8 wt.%			36 dB
[104]	PMMA	MWCNT Uniform diameter: 60–70 nm Length: 50–100 μm	Solvent casting Compression molding	0–10 vol%			40 dB
[105]	Ethylene Methyl Acrylate (EMA)	Ketjen carbon black (K−CB)	Solvent evaporation Hot-pressing	0–20 wt.%	8.6 wt.%	~10^−2^ S/cm	33.9 dB
[106]	EMA	SWCNT Mean diameter: 2 nm Length: 5 μm	Solution mixing Compression molding	0–15 wt.%	1.96 wt.%	~10^−4^ S/cm	44.85 dB
[107]	Paraffin wax (PW)	Nickel-coated carbon nanofiber Average diameter circa: 140 ± 20 nm	Solution mixing Electrospinning calcine	10 wt.%			reflection loss 44.9 dB
[108]	PW	FeNi@ carbon nanofiber Diameter circa: 170 nm	Solution mixing Electrospinning	5 wt.%			reflection loss 31.3 dB
[109]	PW	Ni−Co−coated carbon fiber	Solution mixing Compression molding	30 wt.%		1313 S/m	41.2 dB
[110]	Epoxy resin	Ni−Co alloy−coated carbon fiber Diameter: ~7 μm	Compression molding				75–80 dB
[57]	Silicone rubber	Nickel filaments Diameter: 0.4 μm Length was > 100 μm	Compression molding	3–19 vol%		0.02 Ω·cm	90.5 ± 5.5 dB
[59]	PES	Nickel filaments Diameter: 0.404 ± 0.022 μm	Compression molding	3–19 vol%		10^−2^~10^−3^ Ω·cm	91.7 ± 6.6 dB
[111]	Polylactic Acid (PLA)	MWCNT Diameters: 10–15 nm Lengths: 30–50 μm	Melt blending scCO_2_ foaming Sinter	0–0.0054 vol%	0.00094 vol%	~10 S/cm	50 dB
[112]	Acrylonitrile-butadiene−styrene (ABS)	MWCNT Average diameter: 9.5 nm Length: 1.5 μm	Solution processing Cast	0.5–15 wt.%	0.5 wt.%	1 Ω·cm	50 dB
[113]	Isotactic Polypropylene (iPP) /Poly (ethylene−co−1−octene) (POE) (25/75)	MWCNT Average diameter: 9.5 nm Length: 1.5 mm	Melt blending	~0–3 vol%	0.24 vol%	~0.1 S/cm	~25 dB
[114]	PP/PLA (70/30)	Nickel-coated CF Diameter: 7.0–7.8 μm CNT Diameter: 9–12 nm	Melt blending	0–20 Phr 5 Phr		~10^1^ S/m	50.5 dB
[115]	PS/PMMA (1/1)	MWCNT Mean diameter: 9.5 nm Mean length: 1.5 μm	Melt blending Compression molding Supercritical carbon dioxide (scCO_2_) foaming	0–7 wt.%	0.14 vol%	1.64 S/m	23.08 dB
[116]	PS/PMMA (1/1)	MWCNT Mean diameter: 9.5 nm Mean length: 1.5 μm	Melt blending ScCO_2_ annealing	0–4.58 vol%	0.08 vol%	~10^−2^ S/m	43.73 dB
[117]	PVDF/PS (1/1)	MWCNT Diameter: 8–15 nm Length: 30–39 μm	Melt blending Compression molding	0–15 wt.%	~5 wt.%	~0.1 S/cm	32.99 dB
[118]	PVDF/PLA (1/1)	MWCNT Average diameter: 20–30 nm Average length: 10–30 μm	Melt blending Solution-flocculation method Pressed	0–7 wt.%	0.25 wt.%	~0.01 S/cm	60 dB
[64]	PCL/PS	MWCNT Mean diameter: 9.5 nm Mean length: 1.5 μm	Melt blending Compression molding ScCO_2_ foaming	0–5 wt.%	0.16 wt.%	10^1^–10^2^ S/m	37.4 dB
[119]	PVDF/ethylene−α−octene block copolymer (OBC) (78/22)	MWCNT Average diameter: 10–20 nm Average length: 0.2–2 μm	Melt blending Compression molding	0–9 vol%	2.4 vol%	~0.1 S/cm	~34 dB
[120]	Poly(Phenylene Oxide) PPO/PS (35/65)	CNTs Average diameter: 10−15 nm Average length: 10 μm	Melt-compounding	0–10 wt.%		33.42 S/m	23–25 dB
[121]	Ethylene Vinyl Acetate (EVA)/ UHMWPE (1/4)	CNT Average diameter: 9.5 nm, length: 1.5 μm	Solution-mixture Hot compression	0–7 wt.%	0.014 vol%	~10^2^ S/m	57.4 dB
[122]	EMA/Ethylene Octene Copolymer (EOC) (1/1)	Vulcan XC 72 conductive carbon black (VCB)	Solution mixing	0–30 wt.%	~10 wt.%	~10^−2^ S/cm	31.4 dB
[123]	EMA/EOC (1/1)	MWCNT/VCB (1/1)	Solution mixing	20 wt.%		~10^−1^ S/cm	37.4 dB
